# The Adoption Factors of Mobile Games in the Wireless Environment

**DOI:** 10.1155/2022/7108029

**Published:** 2022-07-01

**Authors:** Bo Li

**Affiliations:** Business School, Central South University, Changsha 410083, China

## Abstract

The factors that influence how people play mobile games have been studied from a variety of perspectives in the wireless broadband environment. The original data in the background of the game, such as user operation records, consumption records, and social behavior records, are converted into user attributes, user tags are generated, and data sets are constructed in this study, which primarily uses data mining technology to study user behavior and form user portraits. By incorporating the similarity of players' subspace interests into the CFR (collaborative filtering recommendation) algorithm, a personalized game recommendation model, as well as the relationship management level of mobile game players, is created. The final fusion model's ROC-AUC value is 0.921, which has a percentile enhancement effect, according to the results. The findings show that using a personalized game recommendation model can help to improve the scalability of the CFR algorithm and the impact of data scarcity on the quality of mobile games recommended by players.

## 1. Introduction

People are increasingly using mobile devices as smartphones become more affordable and mobile data networks improve. Mobile games are a popular leisure activity due to their convenience, portability, and low cost. In the domestic game market, the rate of growth of game users has slowed, and users are showing signs of saturation. The cost of acquiring traffic for game companies is continuing to rise against the backdrop of wireless broadband, but the resulting increase in revenue is unlikely to be proportional. The popularity of smartphones, combined with the ongoing enrichment of mobile game content, has greatly increased users' interest in and experience with mobile games. Mobile games will become a high-growth business sector of the mobile Internet in the future. Measuring and evaluating the quality of the user experience are critical. Displaying abstract user experience in a user-friendly manner can directly reflect the benefits and drawbacks of products, which is useful for product improvement.

With the development of wireless broadband, the scale of mobile Internet is constantly expanding, and the users of mobile game industry are growing rapidly. Ma et al. proposed that the experience economy is being separated from the service economy to provide users with unique experiences. Experience transcends service because it realizes users' self-realization and is free [1]. Brand et al. put forward three quantification methods, namely behavior-centered quantification method, experience-centered quantification method, and task-centered quantification method [2]. Narudin et al. put forward that user experience is the user's experience of brand information, functionality, information availability, and content [3]. Yamamoto proposed that the user experience should include the environment used, the user's perception, and expectation [4]. Jeon et al. expounded the development status of mobile games [5], analyzed the characteristics of mobile games, such as portability, diversified game modes, and rich social interaction, and looked forward to the future development of mobile games. The loss of users can be divided into two situations: one is the lost users, who completely stop trading with the enterprise; the other is potential churn users, who tend to churn but are not completely cut off from the enterprise, but their purchase frequency has decreased, and they will go to their competitors to buy similar products or services.

Mobile games combine the benefits of mobile Internet with game entertainment. This study examines users' willingness to play mobile games and the factors that influence their willingness to play mobile games from the user's perspective using empirical research. A general model of mobile game users' willingness to use is constructed through literature reading and summarization, in combination with the technology acceptance model, opinion leader theory, and related theories, and combined with innovation and communication. Not only will this allow mobile game manufacturers and other content providers to improve their products on a continuous basis, but it will also encourage game operators, game platforms, and other service providers in the game industry chain to do so. More importantly, they have improved the game industry's service level and promoted the industry's development and progress. Innovations in research are as follows: (1) the factors influencing the variables are further explored, and a general model of mobile game users' willingness to use is established, by combining innovative variables such as endogenous value, perceived compatibility, and perceived ease of use. (2) This study divides the item set into several subspaces based on fuzzy clustering based on player behavior situation, considers the proportion of players' common game scores in each scoring scale, and introduces the similarity of players' interest differences in subspaces to improve the calculation of player similarity.

The organizational structure of this study is as follows: the first section introduces the research background and significance and then introduces the main work of this study. The second section mainly introduces the related technologies of user behavior analysis. The third section puts forward the specific methods and implementation of this research. The fourth section verifies the superiority and feasibility of this research model. The fifth section is the summary of the full text.

## 2. Related Work

### 2.1. Research on Mobile Games

The mobile game industry began to develop rapidly, and the number of users increased exponentially. Many mobile game manufacturers have emerged, and mobile games are gradually showing their charm. The entertainment, convenience, and universality of mobile games are more in line with the tastes of fashion people. There are three main reasons behind the rapid development of mobile games [6]: first, the rapid development of wireless broadband and hardware technology [7] has provided a good technical guarantee for the upgrading of mobile games, which has strongly stimulated the market demand; secondly, with the development of economy, the pressure of life is increasing, and the lifetime is becoming more and more fragmented. Mobile games can better fill the fragmented time and bring people psychological relaxation. Third, compared with traditional PC games, mobile games have the advantages of high investment, high return rate, short capital return cycle, and more attractive investment.

Fu et al.'s research shows that the cultural industry, as an important part of China's national economy, has made great progress in the past 40 years of reform and opening-up, with its overall scale greatly expanded, high growth rate, strong stages, and large increase of market players [8]. Duarte et al.'s research also shows that with the help of wireless broadband, a large number of new cultural industries, represented by the electronic economy, have begun to grow and develop, putting forward new requirements for national supervision and policy-making [9]. Liu et al. focused on the deep-seated reasons behind the intellectual property fever of mobile games and discussed the formation law and derivative ability of high-quality intellectual property [10]. Bell pointed out that emotional experience captures the psychology of consumers and makes them deeply impressed by the new products they come into contact with and dominate their own behavior [11]. Li and Bhanu put forward the first game evaluation method, referring to the usability evaluation method and the practical experience of game development companies, and divided the game design into three dimensions: game interface, game mechanism, and gameplay [12]. Takano et al. proposed that the game design is based on the user's interactive experience and the game should include immersion, pleasure, operability, and repeated playability [13]. Yang et al. applied immersion theory to the study of intention to use, and it proved to be effective. With the deepening of research, new theories will be introduced continuously, but the research has never been separated from users [14].

### 2.2. Research on User Behavior Analysis

User preference can be simply understood as the degree, to which a user prefers one thing to another. It is a subjective psychological tendency of users and belongs to the category of psychology. This research has penetrated into the fields of economics and computer science. Li et al. pointed out that user preferences can be simply summarized into two categories: individual preferences and situational preferences. The user's preference model is quantitatively constructed by assigning a certain value to each candidate by using the function [15]. Ye et al. use ontology knowledge to build a complex user preference model based on user text description information [16]. Nowadays, the widely used personalized service system uses the statistical learning method to model user preferences.

For games with a large number of users and complex data attributes, especially online games, data extraction, and utilization are very important. It is a set of behaviors derived from users' psychology. Only through the user's psychology, we can better predict the content of the game mechanism to meet the internal needs and desires, so as to create a successful game. Zhang et al. used a clustering algorithm to segment customers and a genetic algorithm to optimize variables, so as to build a customer lifetime value segmentation model [17]. Sarker et al. respectively selected customer segmentation variables and adjusted variable weights through group decision-making and analytic hierarchy process and then used clustering technology to segment customers of a rubber product manufacturer [18]. Teng and Khong first reduced the dimension of variables, then subdivided four kinds of customers by clustering method, and analyzed the customer characteristics of different subdivided types by the DT (decision tree) method [19]. Zhang et al. used the Bayesian method to calculate the category preference of situational users, and based on context awareness, adopted CFR (collaborative filtering recommendation) algorithm to recommend personalized information to users [20].

## 3. Methodology

### 3.1. Research on Mobile Game Users' Preference

Mobile games are mainly provided by mobile devices such as smartphones and tablets. In mobile games, there is no cost to produce virtual goods, so the more virtual goods players buy, the more profits operators will make [3]. In this study, the perceived usefulness of mobile games is defined as the degree, to which users can enhance the game experience by purchasing virtual goods and other consumer behaviors. The quality of service experience is influenced by many factors, such as personal background, environment, and product itself. Different people will have different judgments on the same product. The quantitative evaluation of user experience can better evaluate the service quality of products, and the evaluation results can also be used to improve products.

Game factors include system quality, service quality, game design, and brand image. The quality of the game system represents the stability, usability, and other factors of the game system, which is the key factor to ensure the normal experience of users when using the game. Service quality can guarantee users' use and ensure users feel comfortable and happy in the process of game experience; the performance of skill and effort games depends on the player's physical and mental state and subjective will at that time, while luck itself is the biggest uncertainty in theory, which may or may not have a certain probability. However, in game design, events are often regarded as a set with a specific probability, and the most interesting thing is the difficulty of the task. Mobile games are entertainment value-added services with the purpose of entertainment. People's increasing work, life pressure, and “fragmentation” time increase the demand for entertainment and leisure, so this study puts forward the variable of perceived entertainment.

**Figure 1:**
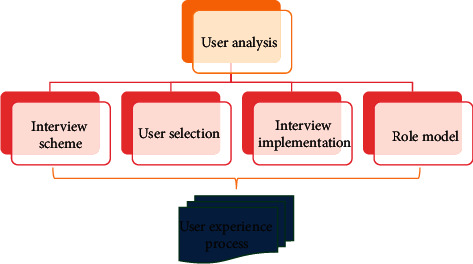
User experience process of mobile game.

User preferences do not remain constant over time; they evolve and change. The preferences of users will emerge, develop, and fade over time. It has qualities of immediacy, randomness, and spontaneity, is easily influenced by the user's situation, is elicited by the attraction of the characteristics of things themselves, and is a type of instantaneous feeling. People are gradually realizing that the quality of personalized service is dependent not only on algorithmic development and improvement but also on the quality of user preference models, which has become a research hotspot and direction. The reasonable acquisition and analysis of user data are the main focus of the user demand analysis of mobile games based on problem situations. The personas are modeled using the interview content, and typical users with common characteristics are created, such as goals and behavioral characteristics. The scenario scripts are created according to modeling roles, so as to easily extract and analyze user requirements. The specific process is shown in Figure 1.

If the *m*-dimensional data set is *X*, the cluster is divided into *k* clusters *w*_1_,…, *w*_*k*_, and their centroids are *c*_1_,…, *c*_*k*_; then,

(1) $$\begin{array}{c} c_{i}=\frac{1}{n}\sum_{x\in w_{i}}x. \end{array}$$

Among them, the number of data points in cluster *w*_*i*_ is denoted as *i* and the number of objects in data set *X* is denoted as *n*.

In the above iteration process, the square error criterion is adopted to judge whether the iteration is finished or not, as shown in the following formula:

(2) $$\begin{array}{c} E=\sum_{i}^{k}\sum_{p\in c_{i}}{\left|p-c_{i}\right|}^{2}, \end{array}$$

where *p* is a point in space, representing a given object.

**Figure 2:**
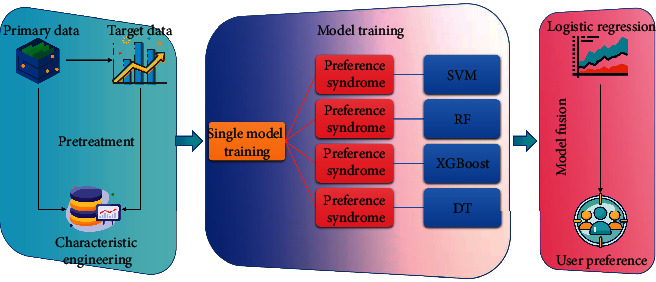
Principle block diagram of the user preference extraction algorithm based on multimodel fusion.

This study simply divides user preferences into positive preferences and negative preferences. At this point, the problem of user preference extraction can be simply understood as a common binary classification problem in machine learning. The SVM (support vector machine) algorithm has a good classification effect when the number of samples is small. Based on the kernel function technique, it can handle nonlinear separable problems well, has strong generalization ability, and can handle high-dimensional data well. XGBoost, like DT, draws on the idea of boosting algorithm. The former has made some improvements on the latter algorithm, and its performance is better. The overall principle of the algorithm is shown in Figure 2.

SVM, RF (random forest), DT, and XGBoost are selected as the basic user preference extraction models. Based on the parameter optimization of single-model user preference extraction methods, the stacking integrated learning framework is used to model the extraction of individual user preferences fusion. The weights of single-model user preference extraction models are obtained through the training of logistic regression method, and the final user preference extraction method is obtained according to the combination of weights.

Logistic regression is a classification model [18], which is expressed by conditional probability *P*(*Y|X*) and formalized as a parameterized logistic distribution. The value of random variable *X* is a real number; that is, the user preference characteristics extracted from each single model, and the value of random variable *Y* is 1 or 0, that is, the positive and negative preferences of users.

Binomial logistic regression is the following conditional probability distribution:

(3) $$\begin{array}{c} P\left(Y=1|X\right)=\frac{\exp\left(w\cdot x+b\right)}{1+\exp\left(w\cdot x+b\right)}, \end{array}$$

(4) $$\begin{array}{c} P\left(Y=0|X\right)=\frac{1}{1+\exp\left(w\cdot x+b\right)}. \end{array}$$

By comparing the two conditional probabilities of users' positive and negative preferences, it classifies users' preferences into the one with higher probability. It can be obtained from the above two formulas:

(5) $$\begin{array}{c} \log\frac{P\left(Y=1|X\right)}{1-P\left(Y=1|X\right)}=w\cdot x+b. \end{array}$$

That is to say, in the logistic regression model, the logarithmic probability of positive preference of output *Y*=1 users is a linear function of input *x*. The closer the obtained linear function value is to positive infinity, the closer its probability value is to linear function value, the closer it is to negative infinity, and the closer it is to zero.

We set the probability of user loss leave=*T* as *p* and the probability of nonloss as *q*, and then the probability of user loss is *α* times higher than that of nonloss under different behaviors, which is OR in logistic regression:

(6) $$\begin{array}{c} \text{log it}\left(p\right)=\text{log}\left(o\;dd\;s\right)=\text{log}\left(\frac{p}{q}\right). \end{array}$$

The log here is equivalent to ln at the base of *e*. Logistic regression is actually an ordinary regression process using logical functions, so

(7) $$\begin{array}{c} \text{log it}\left(p\right)=\text{log}\left(o\;dd\;s\right)=\text{log}\left(\frac{p}{q}\right)=a+bX, \end{array}$$

*X* is a variable, *b* is a coefficient, and *p* is the probability of Class=true, which means the coefficient of logistic regression is defined in terms of log (*odds*)interm of log(*o*  *dd*  *s*) [14].

### 3.2. Construction of Personalized Recommendation Model for Mobile Games

The utility derived from the perceived quality and expected performance of mobile game services is known as quality. The ability to play games on the go is a key feature of mobile game services. It means that users can play mobile games at any time and have complete control over the amount of time they spend playing [4]. Many gamers use video games as a time-consuming stress reliever. This means that the convenience of mobile games as a form of entertainment that is not constrained by time or space has a significant impact on player usage. The difficulty and complexity of users will increase as each link in the process becomes more complex. As a result, in order to measure the perception of global users, this study combines many practical features of the mobile game business in its analysis of perception and usability.

**Figure 3:**
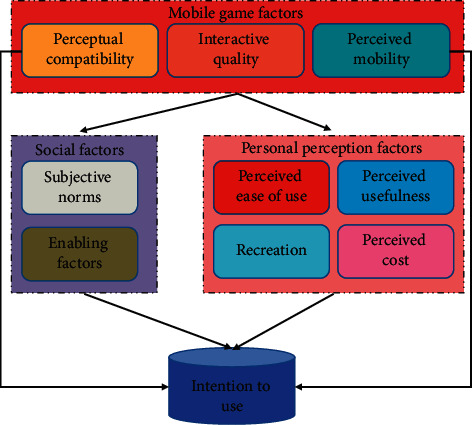
Model of influencing factors of mobile game use intention.

Quality of the system, information, service, game content design, brand image, and other factors all have an impact on game users' participation behavior. Satisfaction is a user's assessment of a product or service's ability to meet their own needs, and it has a positive impact on consumers' purchase intent. Opinion leaders are now inextricably linked to mobile game online marketing. The choices of friends around them will be influenced by the opinions of a group of leaders who are willing to try and spread new games. We can find the influence of opinion leaders in the field of games and the radiation of people through the investigation. In the investigation of this study, enabling factors are expanded into social driving factors according to individual wishes and purposes, such as the promotion of games and a series of marketing strategies adopted by operators. For mobile value-added services, enabling factors are an important means to transform potential users into actual users. Rich data insights can bring higher dimensional data features. When the amount of data is large enough, even if a relatively simple algorithm is selected, better performance can be obtained. In addition, entertainment is the main purpose for users to use mobile games. The greater the entertainment of mobile games, the easier it is to achieve its purpose. Therefore, this study believes that perceived entertainment affects perceived usefulness. The research model of this work is put forward, as shown in Figure 3.

Because these behavioral context attributes of mobile gamers have a certain correlation, this study uses the transitive closure grouping method based on fuzzy equivalence relation to group players. In this study, the ratio of the difference between the number of clusters before and after and the difference between the two clustering thresholds is used as an index to evaluate the clustering quality. The index for evaluating the clustering quality is shown in the following formula:

(8) $$\begin{array}{c} \Delta \delta_{i}=\frac{K_{i}-K_{i+1}}{\lambda_{i}-\lambda_{i+1}}, \end{array}$$

*K*_*i*_ represents the number of clusters for the *i*th time and *λ*_*i*_ is the clustering threshold for the *i*th time. When the threshold value of the *j*th clustering is the maximum value of all clusters and the ratio is the minimum value of all clusters, as shown in the following formula, *λ*_*j*_ is the best threshold value:

(9) $$\begin{array}{c} \left\{\begin{array}{c} \lambda_{j}=\max\left(\lambda_{i}\right)\\ \Delta \delta_{j}=\min\left(\delta_{i}\right). \end{array}\right.. \end{array}$$

This work thinks that the evaluation of the surprise degree of recommendation system should be based on the similarity with users' long-term preferences and the quality of the game itself. As time goes by, preferences are gradually forgotten, and users tend to get a higher degree of surprise. Surprise evaluation of the recommendation results of the whole recommendation system can be expressed as follows:

(10) $$\begin{array}{c} S_{\text{top}-K}=\frac{1}{K}\sum_{k=1}^{K}S_{i,K}, \end{array}$$

where *K* represents the length of the whole recommendation list.

The main process of personalized game recommendation for mobile gamers is as follows:

1. Audit the sample, select the sample player that meets the requirements, eliminate the users with missing or abnormal attribute values, and finally determine the sample users.
2. Calculate the player's interest and fill it into the scoring matrix.
3. The behavior and situational factors of mobile gamers are eliminated, and the original data format is clustered by transitive closure based on fuzzy equivalence relation.
4. In these subspaces, the player game score matrix of the category of the target player is generated. Then, through the improved subspace player similarity calculation formula, the similarity between the target player and all players in the same class is obtained.
5. Get the final player's predicted score value for the unrated game, and recommend the top *N* games with the highest score to the target player.

**Figure 4:**
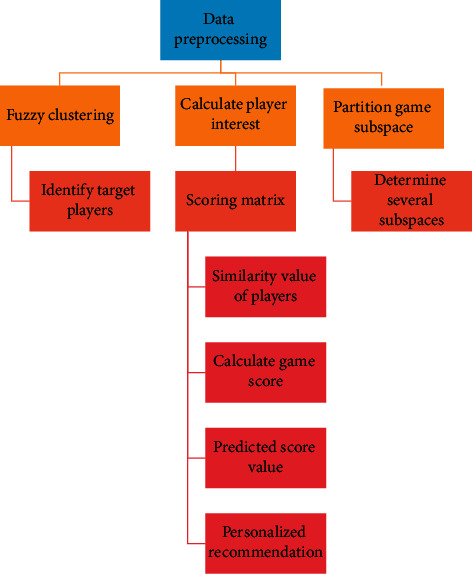
Personalized recommendation process of mobile games.

The personalized game recommendation process is shown in Figure 4.

## 4. Experiment and Results

The mobile game user acceptance model studied in this study has six variables, namely, convenience, ease of use, perceived pleasure, social needs, attitude, and willingness to use. Through the relevant statistical analysis of each model variable, the test results of mobile gamers' acceptance model of combined samples are obtained, as listed in Table 1.

H1–H5, respectively, indicate that convenience has a positive impact on the attitude of using mobile games; ease-of-use has a positive impact on the attitude of using mobile games; perceived fun has a positive impact on the attitude of using mobile games; social needs have a positive impact on the willingness to use mobile games.

As can be seen from Table 1, the *P* value of convenience reaches a significant level of 0.001, and the path coefficient is 0.263, indicating that convenience has a significant positive impact on the attitude of mobile gamers. When players choose mobile games, the convenience of mobile games improves their attitude towards mobile games. The *P* value of social demand reached a significant level of 0.05, and the path coefficient was 0.136, indicating that social demand can have a significant positive impact on mobile gamers' willingness to use. When players choose mobile games, mobile games that meet their social needs will increase their willingness to use mobile games. In the test results, ease of use has a significant negative impact on players' attitudes, but the coefficient is relatively small and the degree of impact is not high.

**Figure 5:**
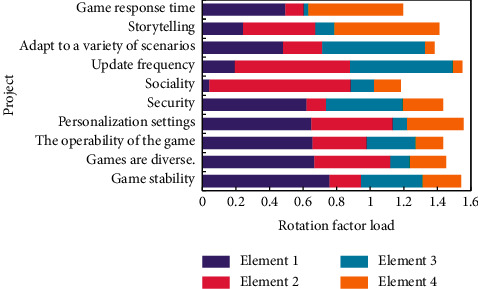
Rotating component matrix.

In this study, SPSS statistical software is used to analyze 180 sample data and 25 categories of mobile game user experience. For KMO and Bartlett tests, the value of KMO test is 0.933, indicating that it is very suitable for factor analysis, while the significance of Bartlett test is 0.000, which has reached a significant level, indicating that the data come from a normal distribution and can be further analyzed. The result of rotating the component array is shown in Figure 5.

It can be seen that the effect of extracting the main factors is that the characteristic value of the first factor is particularly high, which contributes more to the interpretation of the original variables, and the factors that tend to be flat can be eliminated. Among the above assumptions, this study assumes that perceived compatibility, perceived mobility, subjective norms, enabling factors, perceived usefulness, perceived ease of use, and perceived entertainment have a positive impact on intention to use, while perceived cost has a negative impact on intention to use. The relevant analysis results are listed in Table 2.

**Figure 6:**
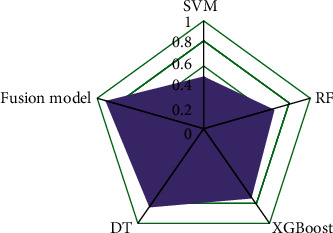
Model performance comparison.

Perceived compatibility, perceived mobility, subjective norms, causes, perceived usefulness, perceived ease of use, perceived entertainment, and perceived cost are all significantly positively correlated with the intention to use. The original hypothesis is accepted because it is negatively related to the clear intention of use. When making predictions, most students will generate a continuous truth value or probability value for the test sample and compare it to a given threshold. It is considered a positive example if the value exceeds the threshold; it is considered a negative example if the value falls below the threshold. Generally, the generalization performance of the classifier is measured by the area under the ROC (receiver operating characteristic) curve, namely, AUC (area under the curve). In this study, ROC-AUC is used as a standard to evaluate the experimental performance, as shown in Figure 6.

**Figure 7:**
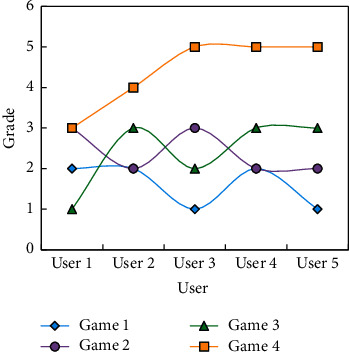
An analysis case of the influence of shadow quality on surprise.

Among them, the user preference extraction results of SVM, RF, XGBoost, and DT in the ROC-AUC evaluation index are 0.501, 0.663, 0.724, and 0.799, respectively. After the stacked models are fused, the ROC-AUC value of the final fused model is 0.921, which has a percentile enhancement effect. In order to further explain the calculation formula of surprise score and its specific meaning and explore the influence of game quality and game similarity and user preference on the surprise score of recommendation system, the following two cases will be discussed. There are 4 games and 5 users in the item rating array, and the default values are filled with hyphens. In order to express the data more intuitively, we plot the data on a line chart, as shown in Figure 7.

It can be seen from Figure 7 that game 1 and game 2 have been scored by user 5, and these scores represent the user's long-term preference information, while game 3 and game 4 have not been scored by user 5. We can not only get the user's preference information but also get the preference similarity information by looking at the trend of the graph. If the line charts of two games have roughly the same trend, then they are similar. That is, the average score of game 4 is higher than that of game 3, which means that the quality of game 4 is higher than that of game 3. This is because when the 3rd and 4th games are the same as the long-term distance of the user 5, game 4 has a higher quality. This case illustrates the importance of game quality in surprise calculation.

**Figure 8:**
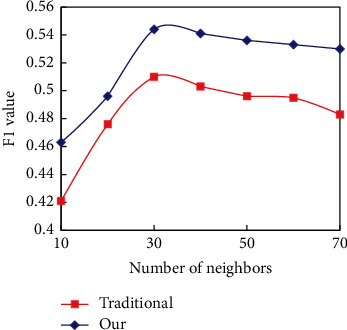
Performance comparison of two methods for calculating player similarity in an 80% training set.

**Figure 9:**
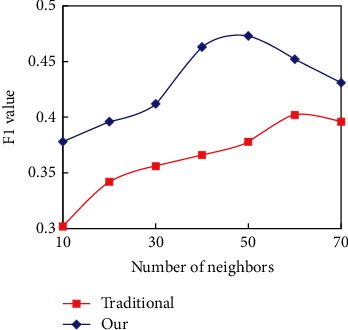
Performance comparison of two methods for calculating player similarity in a 20% training set.

To solve the problem that players are “different” due to data sparseness and alleviate the influence of data sparseness on recommendation quality, an improved subspace player similarity calculation method is proposed, which introduces the interest of players in subspace. The improved player similarity calculation method is compared with the traditional method in two different data sparseness. In terms of setting experimental parameters, the balance weight in the new method of calculating player similarity is 0.8, and the weight of each subspace is equal. The number of adjacent players is selected from 10 to 70, and the recommended number of games is 15. The experimental results are shown in Figures 8 and 9, respectively.

The new method combining the interest differences in player subspace is slightly better than the traditional method in the 80% training set. With the scarcity of data, the difference of F1 index level between the two methods is increasing. Under the training set of 20%, the level difference of F1 indicators produced by the two methods is more obvious. Therefore, a new method of calculating players' similarity by introducing interest differences into subspace can improve the influence of sparse data on CFR quality to some extent by overcoming the problem of “similarity mismatch.”

Through empirical research, it is found that the variable that has the greatest influence on the willingness to use mobile games is the perceived entertainment of games, and entertainment is the highest value attraction point for users to choose to use mobile games. Perceived compatibility, interactivity, and perceived mobility significantly affect the intention of perceived entertainment use. Mobile games should fully consider the different needs of segmented users and design game content according to the entertainment habits and value demands of segmented customer groups. With the accelerated pace of life, people's entertainment time is becoming more and more fragmented, and the demand for light mobile games is increasing. Therefore, game development can balance the two values of entertainment and lightweight and carry out the effective content design.

In the innovation and dissemination of mobile games, light gamers (casual gamers) have less recommendation, less communication, low self-confidence, and their game choices are easily influenced by others, and social channels are easy to reach. Excellent players have rich game experiences, and their game preferences are more consistent in different types of games. Perceived usability and perceived compatibility have limited influence on the reasons why mobile gamers use games, and they are variables that have no significant influence. It shows that the measurement of the above influencing factors is not applicable to mobile games, and mobile game manufacturers need to make other attempts and think about mode planning and promotion.

From the conclusion of the study, it can be found that for mobile game consumers in most areas, whether the game can bring fun or not is the main factor that everyone pays attention to, so it still needs a lot of investment in the future game design. Specifically, mobile games can establish a game community belonging to players in various ways and guide and encourage players to strengthen communication in the community by means of information dissemination, personal status display, and game sharing, so as to gather players with the same preferences. The player's recognition and dependence on the game are enhanced, and the player's sense of identity and participation are enhanced through self-display. At the same time, taking some skillful and complicated operations as the internal design of game level upgrading can not only meet the general users' game experience needs but also produce natural player level differentiation in the game and meet the users' achievement needs.

## 5. Conclusions

In the wireless broadband environment, this research divides the game factors into three main factors: game design, path dependence, and comparative advantage. Using scenario-based theoretical sampling method to conduct interviews, create task models, and scenario scripts, users' needs are analyzed, and preliminary analysis materials are collected. In the process of applying CFR, it is necessary to flexibly adjust the appropriate similarity weight according to the scarcity of data, so as to achieve the best recommendation quality of the algorithm. Experiments show that the new method of calculating the similarity of subspace players can improve the robustness of the algorithm in the case of sparse data, thus improving the impact of sparse data on the performance of the algorithm. In the future, there will be a large number of small models in the game operation, which are more likely to directly become a part of the game, bringing a better game experience to the end users and a long-term beneficial impact on the future development of the game field.

## Figures and Tables

**Table 1:** Hypothesis test result.

Hypothesis	Standardized path coefficient	Test result
H1	0.263	Support
H2	−0.096	Refuse
H3	0.913	Support
H4	0.245	Support
H5	0.136	Support

**Table 2:** Correlation statistics.

Variable	Intention to use	Sig.
Perceptual compatibility	0.566	0.000
Perceptual mobility	0.347	0.000
Subjective norm	0.511	0.000
Contributing factors	0.286	0.000
Perceived ease of use	0.589	0.000
Perceptual entertainment	0.663	0.000
Perceived cost	−0.184	0.001

## Data Availability

The data used to support the findings of this study are available from the corresponding author upon request.
